# Supplementary material for: Predictors of Generalized Anxiety Disorder (GAD) among health care providers during the COVID–19 pandemic at a regional teaching and referral hospital in Western Kenya
Source: PLoS One. 2024 Dec 5;19(12):e0310240. doi: 10.1371/journal.pone.0310240 (PMC11620665; doi:10.1371/journal.pone.0310240)
Supplement: Attachment — Submitted filename: Rebuttal 3 copy.docx [file pone.0310240.s001.docx]

**Response to reviewer 3**

**Please find the response to review concerns in bold**

PONE-D-22-27828R2
Predictors of Generalized Anxiety Disorder (GAD) among health care providers during the COVID – 19 pandemic at a regional teaching and referral hospital in Western Kenya.
Mr Morris Senghor Shisanya

Dear Dr. Shisanya,

We've checked your submission and before we can proceed, we need you to address the following issues:

1. In the Methods section please include the informed consent statement to reflect whether "written or verbal" informed consent was obtained from all participants for inclusion in the study.

**Done: Informed consent statement included to read: “All the respondents provided informed implied consent before participating in the study. Information about the study was provided as a KOBO collect note before starting the questionnaire. Those who clicked “yes” to consent to participating were allowed to proceed. Those who clicked “no” were thanked and exited from the questionnaire.” L162-165**

We've returned your manuscript to your account. Please resolve these issues and resubmit your manuscript within 21 days. If you need more time, please email the journal office at [plosone@plos.org](mailto:plosone@plos.org). We are happy to grant extensions of up to one month past this due date. If we do not hear from you within 21 days, we will withdraw your manuscript.
