# Supplementary material for: Predictors of Generalized Anxiety Disorder (GAD) among health care providers during the COVID–19 pandemic at a regional teaching and referral hospital in Western Kenya
Source: PLoS One. 2024 Dec 5;19(12):e0310240. doi: 10.1371/journal.pone.0310240 (PMC11620665; doi:10.1371/journal.pone.0310240)
Supplement: Attachment — Submitted filename: Rebuttal 4 to editorial comment.docx [file pone.0310240.s002.docx]

**Response to additional editorial comments**

**Please find the response to review concerns in bold**

ACADEMIC EDITOR:

Dear authors

Thank you for submitting the revised manuscript with the changes as suggested by the reviewers and writing detailed responses as well.

**However please add references for the GAD Questionnaire and the SPSS software used and mentioned in the study before the final decision can be taken.**

**Response: Done.**

**Reference for the GAD Questionnaire see LINE 123 (130 in marked copy). Added ref 33** Spitzer RL, Kroenke K, Williams JB, Löwe B. A brief measure for assessing generalized anxiety disorder: the GAD-7. Arch Intern Med. 2006 May 22;166(10):1092-7. doi: 10.1001/archinte.166.10.1092. PMID: 16717171.

**And LINE 133 (140 in marked copy) added ref 33-35**

Spitzer RL, Kroenke K, Williams JBW, Löwe B. A brief measure for assessing generalized anxiety disorder: The GAD-7. Arch Intern Med. 2006;166(10).

Sapra A, Bhandari P, Sharma S, Chanpura T, Lopp L. Using Generalized Anxiety Disorder-2 (GAD-2) and GAD-7 in a Primary Care Setting. Cureus. 2020;

Naeinian MR, Shaeiri MR, Sharif M, Hadian M. To study reliability and validity for a brief measure for assessing Generalized Anxiety Disorder (GAD-7). . Clin Psychol Personal. 2011;9(1).

**Reference for SPSS software added see LINE 151 (161 in marked copy) reference 38**

IBM Corp. IBM SPSS statistics for windows, Version 28.0. Armonk. New York: IBM Corp.; 2021.

Thank you
