# Supplementary material for: Predictors of Generalized Anxiety Disorder (GAD) among health care providers during the COVID–19 pandemic at a regional teaching and referral hospital in Western Kenya
Source: PLoS One. 2024 Dec 5;19(12):e0310240. doi: 10.1371/journal.pone.0310240 (PMC11620665; doi:10.1371/journal.pone.0310240)
Supplement: Attachment — Submitted filename: Rebuttal 5 21.01.2024.docx [file pone.0310240.s003.docx]

**Rebuttal 21/01/2024**

Predictors of Generalized Anxiety Disorder (GAD) among health care providers during the COVID–19 pandemic at a regional teaching and referral hospital in Western Kenya.

**Please find the responses to the Review Comments in the table below.**

| **Reviewer comment** | **Response** |
| --- | --- |
| Reviewer #4: Thank you for inviting me to review this important study. The anxiety among healthcare providers in treating COVID-19 is essential to study.  The study addresses two objectives: 1) to determine generalized anxiety disorder (GAD) and 2) to identify factors associated with GAD. | Thank you for accepting to create time to give us this all important feedback. |
| The study calculates a sample size required to address the first objective, i.e., GAD among providers, but did not calculate the sample size needed to study the associated factors. | **Considered.**  The same study sample population was used to for the two objectives. |
| We see a small sample size for some of the comparisons. For instance, in Table 2, the study had eight Muslims, which is not enough count to compare the odds of GAD. In Table 3, comparing providers with and without COVID vaccination is not valid, with only four providers non-vaccinated. A 95% confidence interval for an odds ratio would be sufficient to identify statistical significance. If an interval includes "1," it will be insignificant and otherwise significant. The p-values could be removed from the tables. | **Considered**  The team chose fidelity to reporting rather than excluding the aspects with cell counts of <5. Fisher’s exact test used to determine association for the cells with < 5 counts as indicated by * on the p values.  The concern about using only 95% CI had been premeditated in the data analysis plan P value to show significance of relationship, OR for strength of relationship with 95% CI showing closeness of our parameters to true population estimation. |
| The study also determines the association using bivariate associations. A multiple logistic regression model would have demonstrated the association of GAD with each variable controlling for the other variables. | **Considered**  This an important observation and the team appreciates that multiple logistic regression or particularly binary logistic regression model would have demonstrated association of GAD with the predictors and controlled for confounders. However, Chi square statistics with ORs from bivariate analysis is a robust test that the team felt achieves the purpose of the study. |
| **Reviewer #5:** |  |
| Firstly, I would like to point out that the authors have done a great job in their work. However; I would like to add a few recommendations: | Thank you for the compliment and accepting to give us this vital feedback. |
| 1. Since the topic is looking for the predictors of GAD among healthcare workers, the study design has the properties of an analytical study. So, I suggest them to modify the study design. | **Corrected**  See L114-116: |
| 2. As the population size is a limited/small size and known, the authors could have taken the total population rather than a sample. | **Considered**  This is an important observation. Conducting a census was initially considered and the hospitals IREC raised concerns as to why conduct a census and not sample part of the staff base. With that considered, the sample size has served the purpose for the study. |
| In addition, they have mentioned and discussed about a formula developed by Fisher and Laing (1998), I believe, it will not add any meaning in their manuscript. | **Considered**  In copy 3 of corrections the academic editor recommended more details on sample size calculation and referencing the sources of the formulae. See rebuttal 3  *“ACADEMIC EDITOR:*  *1. sample size - little more details can be added as to how the sample size was reached including what was the margin of error was taken to estimate the sample size. Please add Reference for the 'n Fisher and Laing 1988' as well. “* |
| 3. Regarding the data analysis, the authors have calculated odds ratios, but they didn’t mention whether it’s from a binary logistic regression or an estimate of Risk Ratio using a chi square test. | **Corrected**  See the table legends.  Table 1 L 188  Table 2 L 208-212  Table 3 L 245-249  Table 4 L 276-280  Table 5 L 304-308  Table 6 L 318-322 |
