# Supplementary material for: Predictors of Generalized Anxiety Disorder (GAD) among health care providers during the COVID–19 pandemic at a regional teaching and referral hospital in Western Kenya
Source: PLoS One. 2024 Dec 5;19(12):e0310240. doi: 10.1371/journal.pone.0310240 (PMC11620665; doi:10.1371/journal.pone.0310240)
Supplement: Attachment — Submitted filename: Rebuttal 6 18.03.24.docx [file pone.0310240.s004.docx]

6. Review Comments to the Author

Please use the space provided to explain your answers to the questions above. You may also include additional comments for the author, including concerns about dual publication, research ethics, or publication ethics. (Please upload your review as an attachment if it exceeds 20,000 characters)

Reviewer #2: Thank you for this opportunity. All comments have been adequately addressed by the authors. I look forward to seeing it in print.

**Response: This is noted. Thank you.**

Reviewer #5: At first glance, the manuscript is well-organized, and the research objectives are clearly aligned with the identified gap in the current literature. In the following comments, I aim to provide constructive feedback to further enhance the clarity, impact, and rigor of the study.

**Response: Much appreciations for the effort to make the manuscript more clearer, impactful and rigorous.**

1. In the previous review of the manuscript, I noted that the authors had not specified which statistical test was employed to examine the relationship between the variables and the outcome. It is crucial that a multiple logistic regression analysis be performed, taking into account all necessary diagnostics, to ensure a comprehensive evaluation of these associations.

**Response: The analysis provided adequately addressed research questions. Adding regression analysis is a good idea but would be an afterthought that was not premeditated in analysis plan at proposal conception.**
2. The authors have provided a lot of tables, I recommend to minimize these tables and only present the important ones.

**Response: Given. The authors deliberately disaggregated the tables to aid the reader in following each of the category of predictors. Putting the content in one table of about 40 rows would be make it a bit tedious for the reader to tease out subtleties in categories of predictors.**
