# Supplementary material for: Predictors of Generalized Anxiety Disorder (GAD) among health care providers during the COVID–19 pandemic at a regional teaching and referral hospital in Western Kenya
Source: PLoS One. 2024 Dec 5;19(12):e0310240. doi: 10.1371/journal.pone.0310240 (PMC11620665; doi:10.1371/journal.pone.0310240)
Supplement: Attachment — Submitted filename: Rebuttal 7 05.07.2024.docx [file pone.0310240.s005.docx]

**Comments to the Author**

1. If the authors have adequately addressed your comments raised in a previous round of review and you feel that this manuscript is now acceptable for publication, you may indicate that here to bypass the “Comments to the Author” section, enter your conflict of interest statement in the “Confidential to Editor” section, and submit your "Accept" recommendation.

Reviewer #6: All comments have been addressed

Reviewer #7: All comments have been addressed

Reviewer #8: (No Response)

2. Is the manuscript technically sound, and do the data support the conclusions?

The manuscript must describe a technically sound piece of scientific research with data that supports the conclusions. Experiments must have been conducted rigorously, with appropriate controls, replication, and sample sizes. The conclusions must be drawn appropriately based on the data presented.

Reviewer #6: Partly

Reviewer #7: Yes

Reviewer #8: Yes

3. Has the statistical analysis been performed appropriately and rigorously?

Reviewer #6: No

Reviewer #7: Yes

Reviewer #8: Yes

4. Have the authors made all data underlying the findings in their manuscript fully available?

The [PLOS Data policy](http://www.plosone.org/static/policies.action#sharing) requires authors to make all data underlying the findings described in their manuscript fully available without restriction, with rare exception (please refer to the Data Availability Statement in the manuscript PDF file). The data should be provided as part of the manuscript or its supporting information, or deposited to a public repository. For example, in addition to summary statistics, the data points behind means, medians and variance measures should be available. If there are restrictions on publicly sharing data—e.g. participant privacy or use of data from a third party—those must be specified.

Reviewer #6: Yes

Reviewer #7: Yes

Reviewer #8: Yes

5. Is the manuscript presented in an intelligible fashion and written in standard English?

PLOS ONE does not copyedit accepted manuscripts, so the language in submitted articles must be clear, correct, and unambiguous. Any typographical or grammatical errors should be corrected at revision, so please note any specific errors here.

Reviewer #6: Yes

Reviewer #7: Yes

Reviewer #8: Yes

**6. Review Comments to the Author**

Please use the space provided to explain your answers to the questions above. You may also include additional comments for the author, including concerns about dual publication, research ethics, or publication ethics. (Please upload your review as an attachment if it exceeds 20,000 characters)

Reviewer #6: With 202 health care providers participating, the sample size appears adequate for statistical analysis. Nonetheless, the study should clarify the sampling technique used to ensure the sample is representative of the larger population of healthcare providers.

**Response: Sampling technique clarified in line L151 to 164: Details on proportionate sampling included**

The authors used Chi-Square statistics to determine predictors of GAD, which is appropriate for categorical data. However, it would be beneficial to include multiple logistic regression analysis to control for potential confounding variables, offering a more comprehensive evaluation of the associations.

**Response: Proposed logistic regression analysis reconsidered. See Lines 176-180 in analysis, and Predictors of Generalized anxiety disorder Line 367-387 and Table 7 in the results**

Odds Ratios (ORs) and p-values are provided, but confidence intervals should also be included to give a range of the estimated effect and its precision.

**Response: Confidence intervals included**: **See Tables 2-6**

Measures to ensure confidentiality should be clearly described, including how data was anonymized and stored securely. This is crucial in maintaining participant trust and ethical integrity.

**Response: Confidentiality and anonymity described in lines L183 – 185**.

The prevalence of anxiety symptoms (59.9%) among health care providers is significant and aligns with global findings. However, the discussion should delve deeper into comparing these results with other regional studies to provide a broader context.

**Response: Done: See lines: L370,371, 375-381, 383-384, 388-394**

The authors should explore potential reasons behind the high prevalence of GAD, such as specific stressors unique to the COVID-19 pandemic and how these compare to pre-pandemic levels of anxiety among health care workers.

**Response: Done: See Lines L396-398, 403-407, 410-414, 435-438**

Reviewer #7: Congratulations for well drafted article. Few suggestions- Objectives of the study could be mentioned.

**Response: Thanks. Objective of the study included in Lines L105-110**

In methodology section, study design would be cross sectional study not analytical. Study duration could be clearly mentioned.

**Response: Considered see L113-115**

Conclusion could be aligned and rephased as per objectives of the study. Sample size (N=?) could be mentioned in each legends of tables or figures.

**Response: Considered see each table and figure and also L531-554 in the conclusions**

Data duplication is there in tables and text, which could be avoided and written in more concise way in the result section.

**Response: Corrected. Prose narration for the tables and figure have removed verbatim reporting to more insightful report of the table.**

The novelty of the study could be justified in the rationale of study at the end of introduction.

**Response: Considered. See L105-110**

Reviewer #8: It is a well written article with good scientific merit. A few changes would enchance the article which are listed below:
1) Kindly provide proper definition of independant variables / risk factors in the study (Eg: Other cadres, previous pandemic?)

**Response: Done see Lines 170-173**

2) Crteria under which social support is categorized into low moderate and high using MSPSS scale could be provided with references.

**Response: Criteria and reference provided. See lines L337-347**

3) How the author has selected 202 from 352 HCP? The author can provide the inclusion and exclusion criteria

**Response: Inclusion criteria added: See lines L148-154**
4) In Figure 1, the data labels could be provided above the bar for improved clarity. Percentage could be mentioned beneath the numbers.

**Response: Figure edited to provide percentages and proper labeling for clarity**

5)Regression models could be provided to enhance the statistical associations.

**Response: Proposed logistic regression analysis reconsidered. See Lines 176-180 in analysis, and Predictors of Generalized anxiety disorder Line 367-387 and Table 7 in the results**

6) The author could avoid repeating the findings in conclusion section.

**Response: Results removed from conclusion section**
